# A novel approach to seabird posture estimation: finding roll and yaw angles of dynamic soaring albatrosses using tri-axial magnetometers

**DOI:** 10.1098/rsos.231363

**Published:** 2023-12-06

**Authors:** Stefan Schoombie, R. P. Wilson, P. G. Ryan

**Affiliations:** ^1^ FitzPatrick Institute of African Ornithology, DST-NRF Centre of Excellence, University of Cape Town, Rondebosch 7701, South Africa; ^2^ Department of Biosciences, Swansea University, Swansea SA1 8PP, UK; ^3^ Centre for Statistics in Ecology, Environment and Conservation (SEEC), Department of Statistical Sciences, University of Cape Town, Cape Town, South Africa

**Keywords:** accelerometer, video camera‌, body angles, directional cosine matrix, dynamic soaring, seabird behaviour

## Abstract

With advances in bio-logging technology, the posture of animals is now commonly described by inertial measurement units, which include tri-axial accelerometers to estimate pitch and roll angles. Many large seabirds use dynamic soaring flight to travel long distances, but this low-cost flight mode results in high centripetal acceleration, which obscures posture derived from accelerometers. Tri-axial magnetometers are not influenced by acceleration and might provide a way to estimate the posture of animals that experience high centripetal acceleration. We propose a new method to estimate the posture of dynamic soaring seabirds using tri-axial magnetometer data, with the assumption that they do not have large pitch angles during routine flight. This method was field-tested by deploying a combination of bio-logging devices on three albatross species breeding on Marion Island, using bird-borne video loggers to validate the roll angles. Validated data showed that the method worked well in most instances, but accuracy decreased when the heading was close to magnetic north or south. Accurate, fine-scale posture estimates may provide insight into dynamic soaring flight and allow estimates of fine-scale tracks using dead-reckoning, not only for seabirds, but potentially for other species where centripetal acceleration limits the use of accelerometers to estimate posture.

## 1. Introduction

**Figure 1.**
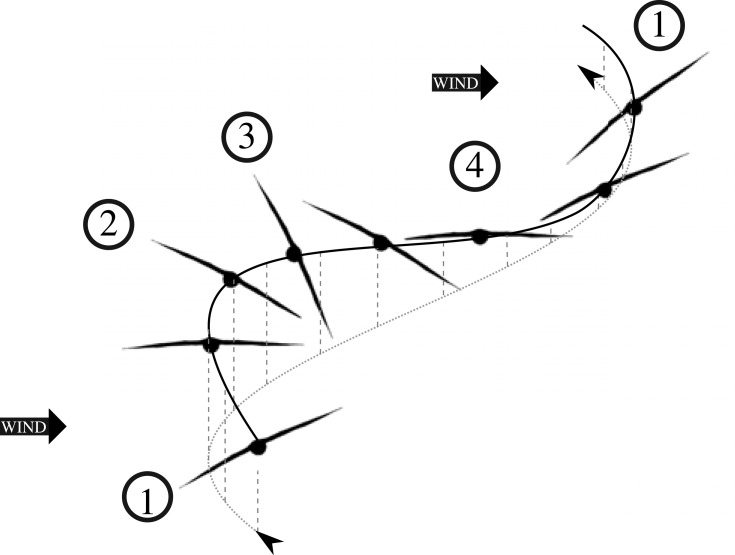
Illustration of a typical dynamic soaring cycle showing the four characteristic elements: (1) a windward climb, (2) a turn to run with the wind, (3) a leeward descent, and (4) a turn to restart the windward climb. The solid black line represents the path of the bird through the air, while the grey dashed lines represent altitude and the grey dotted line the path of the bird over the sea.

The rapidly advancing field of bio-logging has provided researchers with a range of new tools to study animal behaviour [1,2], and particularly so for far-ranging seabirds, such as albatrosses and large petrels (Procellariiformes). These seabirds have a distinct dynamic soaring flight mode, generally consisting of four elements where the bird (1) turns into the wind to gain altitude, (2) turns with the wind, (3) descends with a following wind, and (4) turns into the wind again, restarting the cycle (figure 1) [3,4]. Dynamic soaring allows seabirds to travel large distances while using very little energy [5] and has even been suggested for potential use in the development of unmanned aerial vehicles [6]. With changing wind patterns in the Southern Ocean, it is important to understand how the movement of wind-dependent seabirds may be affected [7,8]. Until recently, the study of dynamic soaring flight was dominated by ship based observations [9–11], but the use of advanced technology has greatly expanded our understanding of this flight action [3,4,12–14]. Fine-scale GPS (or Global Navigation Satellite System) loggers have confirmed that there is a wind-induced propulsive force at the upper turn of the dynamic soaring cycle [13]. Location-based data cannot estimate all body angles of flying birds, but by measuring these angles through other means, we could enhance our understanding of their flight. Inertial measurement units (IMUs) can be used to measure body angles of animals [15] and have the potential to be used when studying dynamic soaring flight [16].

IMUs using accelerometers, magnetometers and/or gyroscopes have been widely used in bio-logging technology [17,18]. Accelerometers are used most often [2,19]; gyroscopes are seldom used due to their high power consumption and sensitivity to environmental conditions [20,21]; whereas magnetometers produce complex data that are often perceived as difficult to use [21]. Another way to assess body posture and movement relies on animal-borne cameras [22], which allows direct observation of behaviour from the perspective of the animal in its natural environment [23]. It is only in the last decade or so that animal-borne cameras have been small enough to use on flying birds [12,23–26]. However, cameras also have high power consumption and record data for shorter durations than IMUs using a similar power source.

Dynamic soaring flight imposes limitations on the use of accelerometers for determining posture and, as a result, accelerometers are not used to study flight behaviour to the same extent as for animals moving on land or in water [21,27]. Tri-axial accelerometers measure instantaneous acceleration in three axes [28]. A stationary accelerometer is subjected to an acceleration of 9.81 m s^−2^ (or 1*g*), as a result of gravity, and the proportion of the 1*g* measured on each of the three orthogonal axes of the accelerometer can be used to derive the animal's posture [15,28]. The static acceleration component of a moving accelerometer can be estimated by calculating the running mean of each accelerometer axis [15]. During flight, many seabirds are subjected to varying centripetal acceleration as a result of their dynamic soaring flight mode [4]. Centripetal acceleration inflates measures of acceleration in the heave axis of the bird, which adds to the static acceleration, and consequently hinders the estimation of posture from accelerometer data [21,27]. Accelerometers can thus only be used to study the posture of slow-moving/stationary animals, or to study short bursts of high energy movement, such as rapid jaw movement when catching prey or individual flaps of flying birds [15]. By comparison, tri-axial magnetometers may be better suited to estimate body angles of flying seabirds, as their measurements are not influenced by dynamic acceleration [21,27]. Nowadays, inexpensive magnetometers are sensitive enough to measure the Earth's magnetic field and tri-axial magnetometers can be interpreted in a similar way as accelerometers to infer posture [15,21]. However, the Earth's magnetic field is not constant, changing in intensity and direction depending on time and location. Fortunately, in recent years, a collaboration between the United States' National Geospatial-Intelligence Agency (NGA) and the United Kingdom's Defence Geographic Centre (DGC) has produced a world magnetic model (WMM), which provides estimates of the magnetic field around the Earth [29]. The spatial data produced by the WMM can be used to correct for regional variations when estimating posture from magnetometer data.

Here, we propose a new method to estimate yaw (*φ*) and roll (*ϕ*) angle from magnetometer data without postural information derived from accelerometers. Our approach is based on assumptions relevant to dynamic soaring flight and requires having at least some information on the location of the animal. The results are first compared to posture estimates from a logger in a controlled environment, where all rotation angles are known and then field tested on wandering (*Diomedea exulans*), sooty (*Phoebetria fusca*) and grey-headed albatrosses (*Thalassarche chrysostoma*) where independent estimates of roll angles were obtained from video loggers deployed with the magnetometers. The control logger allowed us to see how the method performed under varying circumstances and allowed us to highlight potential sources of error. Few studies have been able to estimate the roll angle of dynamic soaring seabirds (e.g. [12,30]) and our novel approach will improve understanding of the propulsive force associated with dynamic soaring and describe possible behavioural responses to changing environmental variables [31]. Our method might also be applicable to other species where dynamic movement limits the use of accelerometers to estimate body angle.

## 2. Methods

### 2.1. Field deployments

A combination of bio-logging devices was deployed on three wandering, one grey-headed and three sooty albatrosses during the 2019/20 breeding season (table 1). Deployments were done on Marion Island (46°S, 37°E) during the brood-guard period in December (grey-headed and sooty albatrosses) and March (wandering albatrosses). The loggers were a combination of customized video loggers (see [30] for details), Daily Diary inertial measurement loggers (DD, Wildbyte Technologies) and GPS loggers (CatTraQ, Catnip Technologies, Ltd; i-gotU GT-120, Mobile Action Technology, Inc.). The video logger deployed on the grey-headed albatross was slightly heavier than the other cameras and consequently we could not deploy a GPS with the DD logger (table 1). DD loggers recorded tri-axial accelerometer and magnetometer data at 40 Hz and were calibrated prior to deployment, as close as possible to the deployment site, to account for hard- and soft-iron offsets in the magnetometry data (following [32] and [21]).The loggers were attached to the birds' back feathers with waterproof Tesa tape (Beiersdorf) in a straight line to minimize drag. The combined masses (including waterproofing and attachment tape) were approximately 130 g for wandering albatrosses and approximately 70 g for grey-headed and sooty albatrosses (less than 3% of body mass [33]) with sampling intervals as shown in table 1.

**Table 1.** Sampling intervals of bio-logging devices deployed on albatross species on Marion Island.

species	*N*	video	DD	GPS	video duration	video interval	GPS interval
wandering albatross	3	x	x	x	continuous	N/A	1 s
sooty albatross	3	x	x	x	continuous	N/A	1 s
grey-headed albatross	1	x	x	—	continuous	N/A	N/A

### 2.2. Analysis

All video footage was inspected and clips where the birds were flying were isolated for analysis (66% of footage; see Results). Subsequently, the corresponding magnetometry data from the DD loggers were also isolated. Data recorded by the video loggers were analysed following [30] where roll angles were estimated from the angle of the horizon in each frame using open source software OpenCV (www.opencv.org) in the Python programming language. Magnetometer data recorded by the DD loggers were used to estimate heading and roll angles from magnetometry data as described below. Throughout the text we follow the notation of [34], but for simplicity we use abbreviated terms: ***h****_i_* is a short notation for a vector in the inertial system and ***h****_b_* the same vector in the body axis system. Here the *xyz* system of the magnetometer is assumed to be the same as the bird body axis system, given that distance between the centre of these two axis systems is small. Likewise, the north east down (NED) system is treated as inertial given that bird flight is close to the surface of the Earth, and rotation of the Earth is negligible at this scale [34]. NED refers to the geographical north, which is in the same plane as the spin axis of the Earth. It is customary in navigation to use lowercase letters for vectors, and uppercase letters for rotation matrices. However, in physics, magnetic fields are typically shown as *H* (uppercase) and because we use terms from both navigation and physics theory, it could become confusing. Hence, for clarity, we retain the uppercase *H* when referring to vectors where ***h****_i_* is rather expressed as *H_i_* and ***h****_b_* as *H_b_*. The initial calculations were performed in the Julia programming language [35] while other analyses were conducted in the R programming environment [36].

#### 2.2.1. Body angle calculation

Tri-axial magnetometers measure the Earth's magnetic field along three axes. When the measured values are compared to the known magnetic field intensity at a given location, they reveal something about the rotation of the measurement device. Such a rotation can be presented as

**Figure 2.**
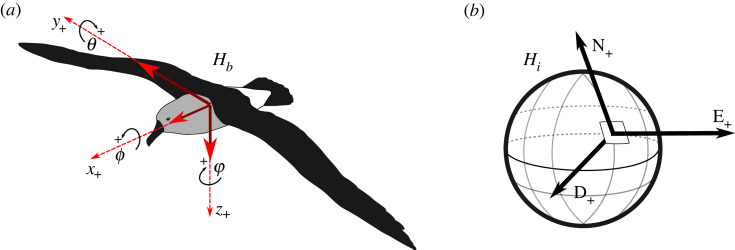
(*a*) Body (*xyz*) coordinate system (dashed lines) applied to an albatross (which is taken as the same as the magnetometer axes, for simplicity), showing the three axes measured by the magnetometers (solid lines) and resultant angles (pitch *θ*, roll *ϕ*, and yaw *φ*) around each axis when compared to (*b*) the north east down (NED) inertial axis system of the reference magnetic field. *H_i_* is a vector with three components projected on the tangent plane axis system of the Earth's surface. North aligns with the longitude line, east aligns with latitude, and down is perpendicular with the tangent plane and aligns with gravity.

2.1 $$H_{xyz}=D_{\mathrm{NED}}^{xyz}H_{\mathrm{NED}},$$

where *H_xyz_* is a vector containing the three values measured by the tri-axial magnetometer, *H*_NED_ is the magnetic vector as given by the World Magnetic Model for a given location [37] (https://www.ngdc.noaa.gov/geomag/WMM/DoDWMM.shtml) and $D_{\mathrm{NED}}^{xyz}$ is the directional cosine matrix (DCM) from the NED axis system to the *xyz* axis system (figure 2). Equation (2.1) can be shortened by calling the *xyz* system *b* (body axes) and the NED system *i* (inertial axes):

2.2 $$H_{b}=D_{i}^{b}H_{i}.$$

**Figure 3.**
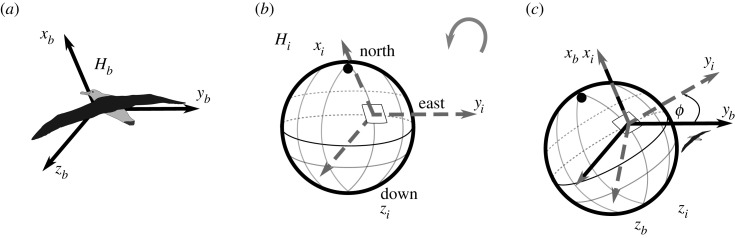
Example of a passive intrinsic rotation around the *x*-axis. (*a*) The body axis system (*H_b_*) stays fixed while (*b*) the inertial axis system (*H_i_*) is rotated in (*c*) where *ϕ* is the roll angle and both pitch and yaw are zero.

There are several ways to represent such a rotation and it is important to note that the following steps are done using a passive intrinsic rotation (figure 3), where the *H_i_* vector is rotated around the body axis system, in a yaw–pitch–roll order. $D_{i}^{b}$ is the resultant 3 × 3 matrix when the rotation matrices around each separate axis (*R_xyz_*) are multiplied with each other (yaw–pitch–roll order), where the yaw, pitch and roll angles are *φ*, *θ* and *ϕ*, respectively.

2.3 $$R_{z}(\varphi )=\left[\begin{array}{ccc} \cos\varphi & \sin\varphi & 0\\ -\sin\varphi & \cos\varphi & 0\\ 0 & 0 & 1 \end{array}\right],$$

2.4 $$R_{y}(\theta )=\left[\begin{array}{ccc} \cos\theta & 0 & -\sin\theta \\ 0 & 1 & 0\\ \sin\theta & 0 & \cos\theta \end{array}\right]$$

2.5 $$\mathrm{and}\;\;\;\;\;\;\;\;R_{x}(\phi )=\left[\begin{array}{ccc} 1 & 0 & 0\\ 0 & \cos\phi & \sin\phi \\ 0 & -\sin\phi & \cos\phi \end{array}\right].$$

When *R_z_* is left-multiplied by *R_y_* and the result is multiplied by *R_x_*, we get

2.6 $$D_{i}^{b}=\;\left[\begin{array}{ccc} \cos\theta \cos\varphi & \cos\theta \sin\varphi & -\sin\theta \\ \sin\phi \sin\theta \cos\varphi -\cos\phi \sin\varphi & \sin\phi \sin\theta \sin\varphi +\cos\phi \cos\varphi & \sin\phi \cos\theta \\ \cos\phi \sin\theta \cos\varphi +\sin\phi \sin\varphi & \cos\phi \sin\theta \sin\varphi -\sin\phi \cos\varphi & \cos\phi \cos\theta \end{array}\right].$$

In equation (2.2), *H_b_* and *H_i_* are both vectors and can thus also be written as

2.7 $$\left[\begin{array}{c} H_{x}\\ H_{y}\\ H_{z} \end{array}\right]=D_{i}^{b}\left[\begin{array}{c} H_{N}\\ H_{E}\\ H_{D} \end{array}\right].$$

Using equation (2.6), equation (2.7) can be expanded to

2.8 $$\left[\begin{array}{c} H_{x}\\ H_{y}\\ H_{z} \end{array}\right]=\left[\begin{array}{c} \cos\theta \cos\varphi H_{N}+\cos\theta \sin\varphi H_{E}-\sin\theta H_{D}\\ (\sin\phi \sin\theta \cos\varphi -\cos\phi \sin\varphi )H_{N}+(\sin\phi \sin\theta \sin\varphi +\cos\phi \cos\varphi )H_{E}+\sin\phi \cos\theta H_{D}\\ (\cos\phi \sin\theta \cos\varphi +\sin\phi \sin\varphi )H_{N}+(\cos\phi \sin\theta \sin\varphi -\sin\phi \cos\varphi )H_{E}+\cos\phi \cos\theta H_{D} \end{array}\right].$$

From equation (2.8) it is apparent that at least two known angles are required per axis to calculate the third angle. Thus yaw, pitch and roll cannot be calculated with only *H_b_* and *H_i_* as known values. However, we can estimate yaw and pitch if we assume that soaring birds do not pitch significantly, which is a reasonable assumption given observations of several albatross species in flight. If the pitch angle (*θ*) = 0, sin*θ* = 0 and cos*θ* = 1, and equation (2.8) can be simplified to

2.9 $$\left[\begin{array}{c} H_{x}\\ H_{y}\\ H_{z} \end{array}\right]=\left[\begin{array}{c} \cos\varphi H_{N}+\sin\varphi H_{E}\\ -\cos\phi \sin\varphi H_{N}+\cos\phi \cos\varphi H_{E}+\sin\phi H_{D}\\ \sin\phi \sin\varphi H_{N}-\sin\phi \cos\varphi H_{E}+\cos\phi H_{D} \end{array}\right].$$

**Figure 4.**
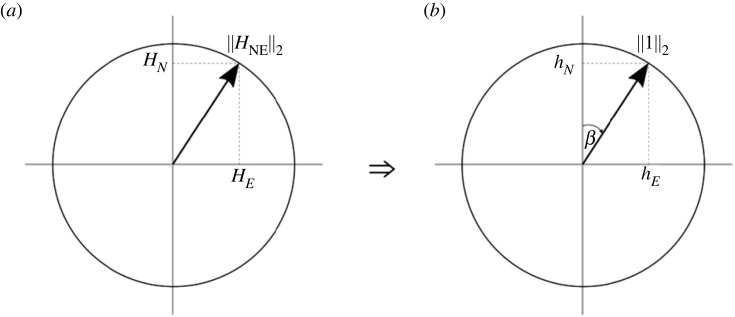
(*a*) The N and E axes of the reference magnetic field are mapped to a unit circle with radius $\|H_{NE}{\|}_{2}$ and (*b*) normalized by dividing by this radius, where *β* will represent the magnetic declination angle.

We can get *φ* from the first row in equation (2.9) and, by substituting *φ* into the second and third rows, we can calculate *ϕ* by solving these equations simultaneously. These equations are solved by first mapping the *H_N_* and *H_E_* values to a unit circle, so that they can be normalized according to the radius of the circle ($\sqrt{\left(H_{N}^{2}+H_{E}^{2}\right)}$ denoted as $\|H_{NE}{\|}_{2}$) and expressed as sine and cosine terms of a single angle (*β*), which is the magnetic declination angle (figure 4).

This angle is chosen to be an angle from the north axis as this is the conventional definition of the magnetic declination angle. Thus, $h_{N}=H_{N}/\|H_{NE}{\|}_{2}$ and $h_{E}=H_{E}/\|H_{NE}{\|}_{2}\;$ so that *β* = tan^−1^(*h_E_*/*h_N_*), ultimately allowing the first row of equation (2.9) to be rewritten as

2.10 $$\frac{H_{x}}{{\left\|H_{NE}\right\|}_{2}}=h_{x}=\cos\varphi \cos\beta +\sin\varphi \sin\beta$$

which simplifies to

2.11 $$\varphi =\pm \cos^{-1}(h_{x})+\beta ,$$

where *h_x_* is the raw magnetometer data from the surge axis (*H_x_*) normalized to the NE plane of the *H_i_* reference data and *β* is the magnetic declination angle at the given location. The loss of sign implied in the cos^−1^ operation is unavoidable, and results in two possible solutions. Once *φ* is obtained, the roll angle *ϕ* can be calculated.

To calculate the roll angle *ϕ*, the same procedure is followed as above with the resultant values from the above calculations substituted into the second and third rows of equation (2.9). Note that *ϕ* is solvable for both the second and third lines of equation (2.9) on their own, but this again results in ambiguous answers. However, if both these equations are solved simultaneously, a single value is returned. Substituting the resultant values into the second and third rows of equation (2.9) simplifies to

2.12 $$H_{y}=-\cos\phi \|H_{NE}{\|}_{2}\sin(\varphi -\beta )+\sin\phi H_{D}$$

and

2.13 $$H_{z}=\sin\phi \|H_{NE}{\|}_{2}\sin(\varphi -\beta )+\cos\phi H_{D}.$$

**Figure 5.**
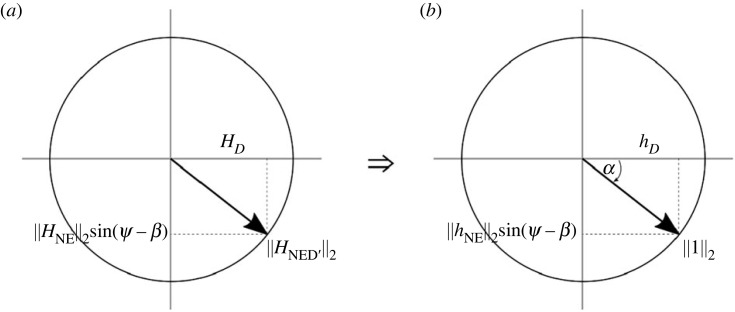
(*a*) Representation of the unit circle where the axes *H_D_* and $\left\|H_{NE}{\|}_{2}\sin(\varphi -\beta \right)$ have a radius $\|H_{NED^{\prime }}{\|}_{2}$ and (*b*) normalized by dividing by $\|H_{NED^{\prime }}{\|}_{2}$, where *α* is measured from *H_D_*. Note that the bottom hemisphere is positive due to the NED axis convention.

For both equations (2.12) and (2.13), $\left\|H_{NE}{\|}_{2}\sin(\varphi -\beta \right)$ and *H_D_* can be mapped to a unit circle, and the radius used for normalization $\sqrt{(H_{N}^{2}+H_{E}^{2})\sin^{2}(\varphi -\beta )+H_{D}^{2}}$ here will be referred to as $\|H_{\mathrm{NE}D^{\prime }}{\|}_{2}$ (figure 5).

This time the angle *α* is calculated from the *z*-axis (positive down axis) so that

2.14 $$\alpha =\tan^{-1}\left(\frac{\left({\left\|H_{NE}\right\|}_{2}\sin(\varphi -\beta )\right)}{H_{D}}\right).$$

Now, when *H_y_* (sway axis) and *H_z_* (heave axis) are normalized by $H\|{\;}_{NED^{\prime }}{\|}_{2}$, the resultant equations are

2.15 $$\frac{H_{y}}{{\left\|H_{NE}\right\|}_{2}}=h_{y}=\sin(\phi -\alpha )$$

and

2.16 $$\frac{H_{z}}{{\left\|H_{NE}\right\|}_{2}}=h_{z}=\cos(\phi -\alpha ).$$

And finally, by dividing *h_y_* by *h_z_*, the roll angle can be calculated by applying a four-quadrant tan^−1^ operation while preserving the sign:

2.17 $$\phi =\tan^{-1}\left(\frac{H_{y}}{H_{z}}\right)+\alpha ,$$

where *α* is a function of *φ* and the reference magnetic field data *H_i_* (equation (2.14)). The loss of sign in the cos^−1^ operator (equation (2.11)) results in two solutions of yaw (and consequently roll) and a *post hoc* decision has to be made to determine which of the solutions is correct. To do this for dynamic soaring albatrosses, we looked at the range of the roll angle values over 10 s (approximate duration of a dynamic soaring cycle). Here, the correct solution will be the one where the sum of the minimum and maximum roll values (for a given 10 s period) is the smallest. This is because the roll angles of dynamic soaring albatrosses must cross zero degrees, within a given cycle, when they turn in in relation to the wind. Thus, the solution with the lowest average roll angle will be the correct one for an individual dynamic soaring cycle. Importantly, the accuracy of this method relies critically on the quality of the calibration and how well the hard- and soft-iron offsets are accounted for.

#### 2.2.2. Validation

To determine how well *ϕ* estimation from magnetometer data worked, the estimated values were compared to *ϕ* derived from tri-axial accelerometer data on a control DD logger (*DD*_0_):

2.18 $$\mathrm{Accelerometer}\;\phi =\tan^{-1}\left(\frac{{\mathrm{Accelerometer}}_{y}}{{\mathrm{Accelerometer}}_{z}}\right).$$

The *DD*_0_ logger was calibrated in the same manner as the loggers deployed on albatrosses. Then, a compass swing was performed, rotating *DD*_0_ at 10° yaw angles (starting at magnetic north) and rotating the logger around the *x*-axis to get roll at each yaw increment (obtained from a compass). These rotations were performed by hand and rotations on the *DD*_0_ logger were undertaken slowly to ensure that the centripetal acceleration was minimal and thus roll angles derived from the accelerometer values could be used as a control. To estimate the effect that small pitch angles may have on the calculation of roll from §2.2.1, we simulated the error at all values of yaw and roll (at 1° increments) when using different pitch values ≠ 0. This was done by first calculating the expected magnetometer values (*H_b__*_expected_), by substituting values of *H_i_* (extracted from the WMM using Marion Island's location, 37°E, 46°S) and the chosen yaw, pitch, and roll angles into equation (2.8). Using the *H_b__*_expected_ values we then calculated the roll angles (assuming pitch = 0; §2.2.1) and report the roll angle error as the difference between the input roll angle and the calculated roll angle. All of the control logger experiments were performed on Marion Island.

#### 2.2.3. Field deployments

The DD loggers each contained a tri-axial magnetometer which produced data used to estimate yaw *φ* and roll *ϕ* angles using the method described above (§2.2.1). When the birds were moving in the same direction as the magnetic field (magnetic north), *φ* and subsequently *α* values could not be calculated (*h_x_* > 1 or *h_x_* < −1) and *ϕ* angles were interpolated by using the previous valid *α* value and substituting it into equation (2.17). These analyses were done using data collected from three albatross species (table 1) where priority was given to recording longer periods of flight with enough overlap between video and DD logger data for validation of the results. The roll angles estimated from video data were compared with roll angles estimated from the magnetometry data to validate the efficacy of the method. This was done by comparing all points corresponding to each five-minute video bin, within individual flights.

Analyses of albatross data were restricted to individuals where enough high-quality data were present from both tri-axial magnetometers and video loggers. For equipped birds, reference roll angles were obtained from video where the horizon angle was used as a proxy for roll angle [30]. The magnetometer data were down-scaled to fit the lower sampling frequency of the video data (24 Hz) and were matched temporally to the video data. The video data were recorded in 5-min video files (bins) and time synchronization between video and magnetometry data was confirmed by visual inspection for each bin. This resulted in a dataset of magnetometer inferred and video observed roll angles at 24 Hz. The error for each inferred roll angle was calculated by subtracting the magnetometry estimate from the video estimate and mean error of the absolute values was calculated for each 5-min video bin for each section of flight across all individuals. In addition, the number of dynamic soaring cycles (identified by consecutive roll angle peaks) was counted from both the video and magnetometer data. These peaks were the maximum roll angle value in between points where roll crossed zero. To determine the importance of location accuracy when extracting reference magnetic field data from the WMM, roll estimates supplemented with tracking data were compared to roll estimates made using a single coordinate, the deployment site on Marion Island. This was done for the flight tracked furthest from the breeding island for each tracked individual.

## 3. Results

All the deployed loggers were recovered, and good quality data were obtained from two wandering albatrosses (five flights lasting 7.0 h), two sooty albatrosses (nine flights, 4.8 h) and one grey-headed albatross (six flights, 2.8 h) that allowed comparison of video- and magnetometer-derived roll angles. The DD loggers recorded an order of magnitude more data (213 h) than the video loggers (22 h flying and on water).

### 3.1. Control logger (*DD*_0_)

**Figure 6.**
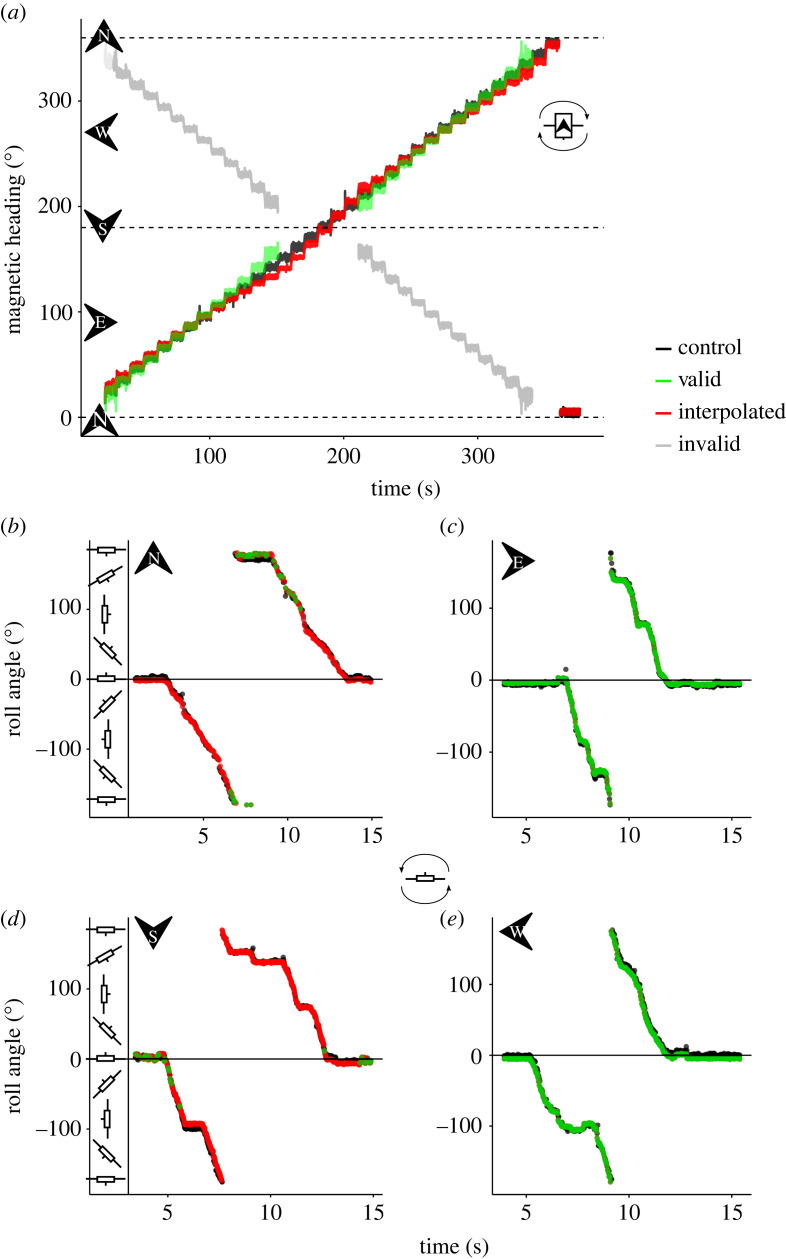
Control logger *DD*_0_ results showing performance of the newly described method for estimating yaw and roll from tri-axial magnetometer data. (*a*) The two solutions of yaw (see equation (2.11)) showing valid (green) and invalid (grey) angles when performing a compass swing of magnetic heading at 10° increments for constant roll angle (0°). Magnetic north and south are indicated by dashed lines. (*b–e*) Roll angle estimated from *DD*_0_ data at varying magnetic headings (north, east, south, and west) while performing a full rotation of roll angles. Black lines are control angles from accelerometer data compared to valid (green) and interpolated (red; using the closest *α* value) magnetometer angles.

**Figure 7.**
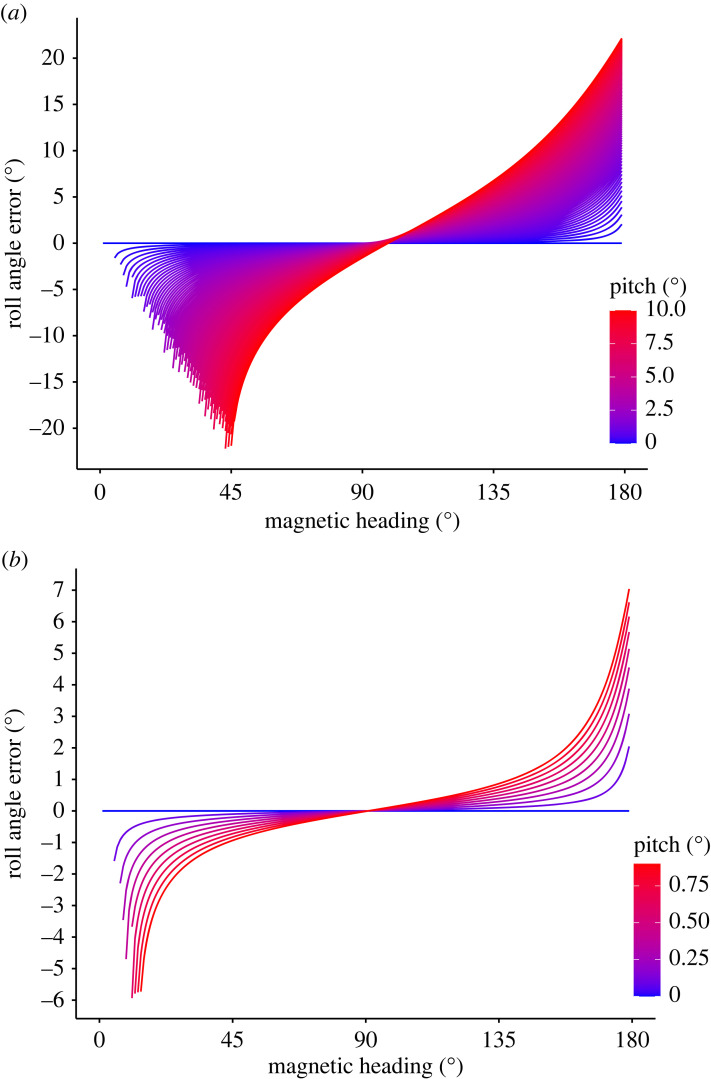
Simulated roll angle error at incremental values of yaw and pitch and roll. (*a*) Roll angles were estimated from simulated magnetometer readings (*H_b_*) at controlled increments of yaw, pitch and roll. The estimated roll angles were compared to the control roll angles to calculate the error. (*b*) A zoomed in section of (*a*) showing only small pitch values (less than 1°). Note that only one hemisphere (heading 0°–180°) is shown as the results are duplicated on the other hemisphere and not shown here.

Roll and yaw angles could be estimated for most headings (magnetic heading), with some gaps around magnetic north and south (figure 6). Yaw angles around magnetic north and south could not be estimated as the absolute *h_x_* values were greater than 1 (when the majority of the magnetic field is measured on a single axis), which is not a valid input for the cos^−1^ operator. Missing roll angles (where *φ* was not available to calculate *α*) were estimated by using the nearest valid *α* value (figure 6). When compared to the roll angles estimated from accelerometer data (regarded as the ‘true’ rotation) a mean error of 4.5 ± 5.5° was found. Roll angles were successfully estimated from the magnetometer data recorded on the *DD*_0_ logger for all headings (10° increments from 0° to 360°), but interpolation (using the previous valid *α* value) was necessary when headings coincided with magnetic north and south (figure 6). Simulated data showed that pitch angles of the order of 10° resulted in roll angle errors up to 20° and reduced the window around the magnetic poles in which valid yaw (and subsequently roll) angles could be calculated (figure 7*a*). However, with small pitch angles (less than 1°) the error in roll angle remained relatively low (less than 5°; figure 7*b*). Roll angle error at varying pitch angles was lowest when the yaw value was closest to magnetic east and west (figure 7).

### 3.2. Roll angle validation

**Figure 8.**
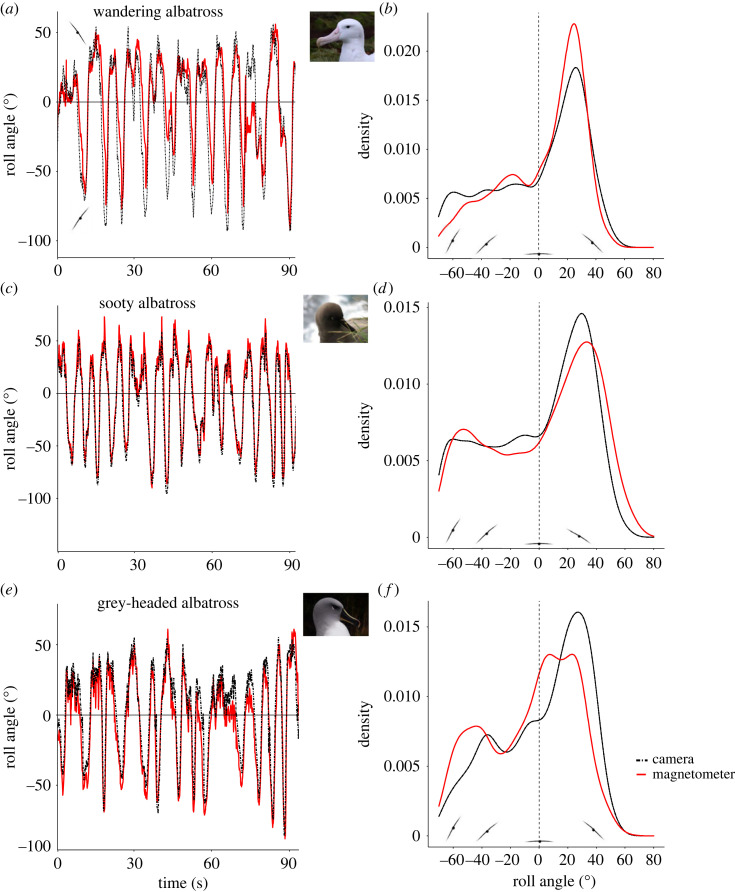
Examples of roll angle estimates from magnetometer data (red lines) for three albatross species (wandering, sooty and grey-headed albatross). (*a,c,e*) Roll angles from a 90 s flight section. (*b,d,f*) Density plots of roll angles for respective species. Control angles were estimated from video loggers (black dashed line) deployed with the magnetometers.

**Figure 9.**
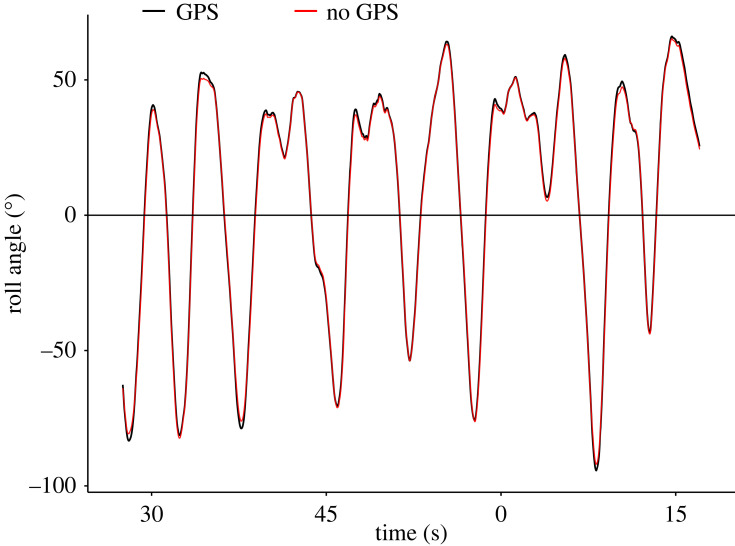
Roll angles extracted from a 90 s sooty albatross flight approximately 330 km from Marion Island. Roll angles were estimated using reference magnetometer data at GPS locations (5-min interval; black line) and using reference data from a single coordinate (Marion Island; red line).

Magnetometer-derived roll angles compared well to those derived from video loggers for all three albatross species, with a mean ± s.d. roll angle error of 15 ± 5° (table 2 and figure 8). This indicates that our assumption of low overall pitch angles during dynamic soaring seems plausible. From the three species, the error was largest for the two sooty albatrosses (18 ± 14°; table 2). It has to be noted that the presented error is not absolute, but rather relative to estimates from video data, which have their own measurement error [12,30]. Location accuracy did not have a large effect on the reference magnetic field within the range of the four individuals tracked with GPS loggers (maximum distance from Marion Island 300–500 km). The mean ± s.d. error for flights at the maximum distance from the breeding island (last flights per individual; table 2) was 1 ± 1° for both roll and yaw angles estimated from magnetometry data (figure 9). The grey-headed albatross data did not include GPS tracking, and a single coordinate (Marion Island; 37°E, 46°S) was used for all magnetometer estimates, which still produced acceptable results (figure 8*e*).

**Table 2.** Error estimates (degrees) of roll angles across individual flights derived from magnetometry (Mag) compared to roll angles from video loggers deployed on three albatross species. All values are degrees (−180° to 180°) unless otherwise stated.

bird ID	flight (number)	duration (min)	mean roll error ± s.d.	roll angle range		dynamic soaring cycles	
				video	Mag	video (*n*)	Mag (*n*)
**wandering albatrosses**							
WA1	1	109	13 ± 12	−84–57	−82–68	602	591
WA1	2	50	15 ± 13	−100–60	−88–59	366	362
WA1	3	45	16 ± 13	−99–58	−89–53	363	353
WA2	1	120	9 ± 9	−93–46	−88–55	715	722
WA2	2	94	10 ± 11	−93–46	−88–52	627	637
**average**			**13 ± 11**				
**sooty albatrosses**							
SA1	1	106	12 ± 12	−96–63	−104–86	876	893
SA1	2	50	13 ± 13	−78–60	−84–84	415	410
SA2	1	8	19 ± 15	−100–64	−94–95	66	63
SA2	2	2	26 ± 19	−88–84	−66–87	15	15
SA2	3	5	24 ± 14	−96–68	−100–83	36	33
SA2	4	49	19 ± 14	−93–71	−92–94	261	245
SA2	5	46	21 ± 15	−85–75	−100–94	213	200
SA2	6	5	19 ± 15	−90–79	−121–114	30	30
SA2	7	19	13 ± 12	−79–107	−79–112	163	162
**average**			**18 ± 14**				
**grey-headed albatross**							
GA1	1	75	11 ± 9	−85–78	−93–86	601	597
GA1	2	38	12 ± 10	−88–78	−102–82	325	327
GA1	3	13	13 ± 13	−88–89	−103–76	98	100
GA1	4	5	13 ± 13	−90–86	−120–91	38	38
GA1	5	35	13 ± 11	−92–80	−110–90	271	270
GA1	6	4	15 ± 14	−90–88	−93–76	35	35
**average**			**13 ± 12**				

**Figure 10.**
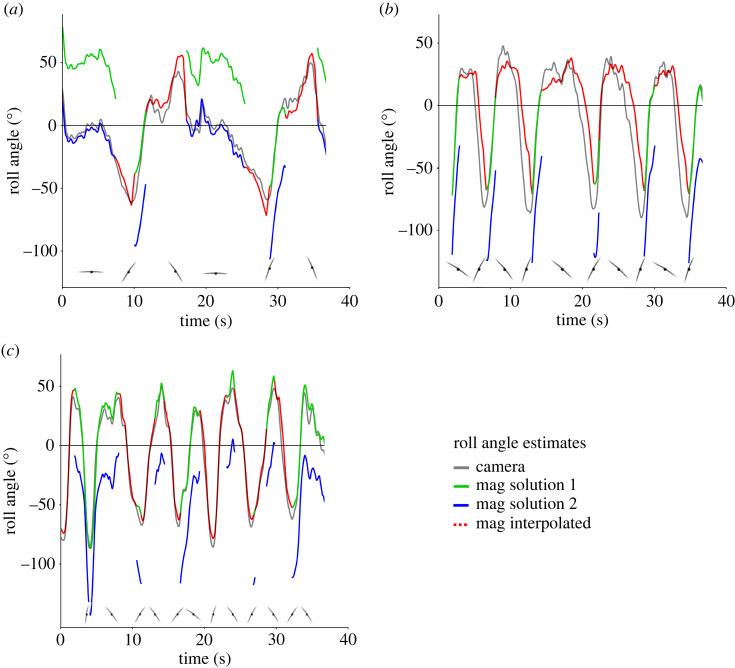
Examples of roll angle estimates from magnetometer data under varying circumstances. The two possible solutions from equation (2.11) are shown (green and blue lines) with interpolated values (red lines) where no valid solution was available. (*a*) Irregular dynamic soaring cycles where both solutions produce correct values. (*b*,*c*) Regular dynamic soaring cycles with valid roll angles mainly following the upper turning phase (*b*) or valid angles present in parts of all phases (*c*), with only one correct solution.

Results from the control logger showed that our method should produce the best results when the birds were not heading directly into magnetic north or south. Likewise, errors seemed lowest when consecutive dynamic soaring cycles occurred, allowing for easy discrimination between the two possible solutions from the yaw angle estimates (equation (2.11)). The solution with the lowest mean value over 2–5 s (half a dynamic soaring cycle) was assumed to be correct as roll angle regularly crosses zero during dynamic soaring (figure 10). Larger errors occurred when the birds were flying with a heading that was close to magnetic north or south for large portions of the flight. As shown above for the control logger, this resulted in *h_x_* values greater than 1 where yaw angles (and subsequently roll angles) could not be calculated. Interpolating the missing values by using the nearest valid yaw value to calculate the roll worked well in most cases, maintaining the shape of the soaring cycle (figure 10). The direction of the roll (positive versus negative banking) was also mostly preserved, even when several seconds of flight path had to be interpolated (figure 10). Another problem that arose with headings that corresponded with magnetic north or south was that the choice between the two solutions for yaw angles (equation (2.11)) became less apparent (figure 10*a*). Even so, the overall shape of the dynamic soaring cycles inferred from magnetometer data corresponded well with the video-derived data (figure 8). When estimating the number of dynamic soaring cycles in a flight, the magnetometer data were just as effective as the video data, with 98.6% of the video cycles identified by magnetometer data (table 2). We did not have validation data for yaw angles (i.e. heading) and thus could not directly assess the accuracy, but seeing that roll angle calculation was dependent on having yaw angle first, it would be fair to assume that the accuracy of yaw was similar to that of roll angles.

## 4. Discussion

Estimating roll angles from magnetometer data is valuable in providing a new means of studying dynamic soaring flight. Magnetometers have been used to estimate the average bank angle of *Gyps* vultures while soaring in thermals [38], or to identify behavioural states from a range of species in both terrestrial [39] and marine environments [16,40,41], but instantaneous measurements of body angles are usually limited to heading for dead-reckoning studies [42] (but see [21]). Studies of avian flight that use IMU technology are mostly limited to the identification of flapping flight [27]. Accurate estimates of roll angles could shed new light on our understanding of dynamic soaring flight, as it is an integral part of this flight mode [3,4,12,43]. It has been hypothesized that the main source of energy gain in the dynamic soaring cycle is a wind-driven propulsive force at the upper turn of the cycle [13,14]. This propulsive force would be dependent on the angle of the bird's wing in relation to the wind (similar to a sailboat; see fig. 2 in [44]), as well as the heading of the bird in relation to the wind [13], and knowing the roll and yaw angles could allow further quantification of such a propulsive force. We present a new method for estimating roll and yaw from magnetometer data alone but acknowledge the obvious difficulty of assessing the errors due to the inherent problems of measuring these angles in the first place.

Bird-borne cameras provide reliable estimates of roll angles for fine-scale analysis of seabird flight behaviour [12,30]. However, video loggers are often constrained by short lifespan and many hours of footage may be spent filming other behaviours, such as preening or resting. IMUs are smaller and can record data for longer periods than video loggers, with the potential to deliver information from entire foraging trips when reliable methods are available to infer body angles. Our method successfully inferred body angles from magnetometer data, and these angles were validated by comparison with data from video loggers. A substantial amount of flying footage was obtained with the filming intervals used in the present study, but the magnetometers produced an order of magnitude more data than the video loggers. Ours is the first study to estimate the fine-scale roll angle of dynamic soaring albatrosses directly using magnetometry data. However, it must be noted that we did not quantify the effect of heading on roll error as we did not have a means to verify our heading estimates. We also did not have a reliable way of identifying ‘consecutive dynamic soaring’, but this may now be possible as a potential method of identifying dynamic soaring has recently been proposed [12]. Further study is needed to establish the absolute error of the roll angle estimates, potentially by using gyroscope data [45], but our approach could be used to study changes in roll angles at various stages of the dynamic soaring cycle, and to assess how this changes with environmental variables [31]. We synchronized the video and accelerometer data manually, which could introduce errors if there is a temporal mismatch between tags [46] and could also contribute to some of the error between video and magnetometer estimates. Sequential video frames (at 24 Hz) rarely had a difference in roll angle greater than 5°, and thus a temporal mismatch of 100 ms could introduce an error of up to 10°, and could be a contributing factor to our error rates. When comparing the distribution of roll angles from video and magnetometer estimates, there seemed to be an offset for grey-headed and sooty albatrosses. It is possible that this offset was caused by a slight mismatch of the tag orientation, where the two tags (camera and DD) were not exactly aligned. This highlights the need for careful consideration of tag placement when body angles are estimated (also see [47]).

By assuming that dynamic soaring albatrosses do not routinely pitch at large angles during flight, we were able to extract yaw and roll angles from magnetometer data for three albatross species. Formally we assume *θ* = 0°, but we believe that the small deviations from the pitch angle assumption that our study albatrosses exhibit are inconsequential for estimating albatross roll and yaw. Albatrosses observed flying around their breeding colonies or out on the ocean seldom pitch significantly (personal observations). This is also evident from the video data, where the horizon is visible in most frames, allowing the extraction of roll angle from the horizon. Our simulated data suggest that small pitch values (up to 1°) did not result in errors > approximately 5°. Higher pitch values (greater than 1°) decreased the size of the window (i.e. proximity to magnetic north and south) where valid *α* values could be calculated, and accuracy of roll angles was lower when yaw was in the proximity of magnetic north and south. However, when yaw was close to magnetic east and west, roll angles were seemingly unaffected by changes in pitch angles. Dynamic soaring birds routinely change direction during flight (e.g. [44,48]) and we can assume that a bird will have a heading close to magnetic east or west for at least part of the cycle. Thus, the magnetometer data should be valid to estimate roll at least once in a cycle and missing angles can be interpolated. The error rates were highest for the two sooty albatrosses, the smallest of the three study species. It is possible that smaller dynamic soaring birds pitch more during flight [12], which could introduce larger error when estimating roll from magnetometer data. We also did not take wind into account, where albatrosses seem to roll at more extreme angles when flying in stronger winds [31], and it is unclear if these large roll angles could be coupled with an increase in pitch as well.

Fine-scale location data were available for all but one bird used in the magnetometer validation study (the grey-headed albatross). Where tracking data were not available, the deployment location was used to determine the local magnetic fields and the inferred roll angles still produced acceptable results. The amount of location data necessary to extract body angles of dynamic soaring seabirds using magnetometers will depend on the species, location and the questions that are being asked. For example, birds that stay close to their breeding colony for an entire foraging trip will not require additional location data, but birds that traverse ocean basins will need at least some location estimates. The frequency of location data needed will also depend on the range of the bird as the magnetic declination becomes increasingly variable closer to a dip pole (where magnetic inclination is 90°), which in the Southern Hemisphere is located at approximately 65° S, 140° W. Where accurate heading estimates are necessary, more frequent location data (e.g. hourly GPS fixes) are appropriate, but coarse location data (e.g. daily estimates from geolocators) should provide sufficient accuracy to estimate roll angles within a dynamic soaring cycle.

Accelerometers are effective tools to study animal movement but cannot be used to infer the headings adopted during these movements. Using magnetometers to determine the direction of movement of an animal [49] and the ability to measure instantaneous heading at high resolution is thus a valuable tool for studying fine-scale animal behaviour. When accurate heading estimates are known, fine-scale movement patterns can be inferred by dead-reckoning [28,32,50]. Dead-reckoning has been used to study long distance routes of flying birds [51,52]. These days, satellite tracking can provide accurate tracks of seabirds' movement spanning days to months, depending on sampling intervals and battery size or power supplementation from solar panels [53]. Satellite tracking has been used extensively to study the flight behaviour of dynamic soaring seabirds [5,6,14,54,55], but these studies are limited to linear interpolations between successive fixes (displacement), the precision of which depends critically on the sampling interval. Dead-reckoning from magnetometer heading estimates can produce tracks at much finer scales (greater than 100 Hz) when speed of movement can be inferred. Increasing the frequency of heading estimates increases the accuracy of dead-reckoned tracks and can shed light on the decisions an animal makes prior to certain behaviours [56]. Further study is required to validate the accuracy of the heading estimates from magnetometer data (present study) as Kempton *et al*. [12] found that heading inferred from video roll angle did not provide sufficient accuracy to perform dead-reckoning in dynamic soaring shearwaters, and a correction factor needed to be applied to their data to account for rotation of the device relative to the bird's body axes. Such a correction may be necessary when our heading estimates are used for dead-reckoning, but this would require validation of the heading estimates from a known source. This could potentially be done with video data if the sun is visible in a frame and the time and location is known [12], but this is not something we explicitly tested here. Although satellite trackers can now be used on even smaller seabird species, the use of magnetometers to estimate tracks might be a better alternative due to their low power consumption. GPS tracks can give information regarding the course over ground, which is not the same as heading when a bird flies in wind with a component across the bird's air velocity direction. The heading of a dynamic soaring bird in relation to the wind is important when studying its flying behaviour, but this information is rarely available.

### 4.1. Limitations and possible solutions

When estimating rotational angles from magnetometer data using a directional cosine matrix, there are several limitations to consider before it is applied to any study. First, this method is only applicable to species where it can be assumed that the pitch angle is negligible. Second, the cos^−1^ operation in equation (2.11) will only give an answer when *h_x_* is between −1 and 1. Outlying values can be the result of electronic noise or bad calibration and will result in the inability to calculate yaw and consequently roll angles. Fortunately, data gaps that result from such outliers may be estimated by assuming that *α* does not change significantly within a short period of time. Then, roll angles can be calculated by substituting *α* from previous reliable estimates into equation (2.17). Third, if both *H_y_* and *H_z_* are zero (yaw coincides with magnetic north) the outcome of equation (2.17) is undefined, which will also be the case on the magnetic equator where this method will not work. In general, whenever a bird is flying in the same direction as the magnetic field, the entire magnetic field is only measured on the *x*-axis and it becomes impossible to infer roll angle. Finally, the cos^−1^ operation in equation (2.11) will result in two valid solutions (and consequently two solutions for *ϕ*), but only one of them will represent the rotation of the magnetometer axes. There are two ways to determine which of the solutions is correct. First, if positional data are available (e.g. GPS positions), the average yaw angle can be compared to the average heading for a specific section of flight to determine which is correct (bearing in mind the effects of wind drift). Second, if the flight behaviour of a study species results in frequent changes in roll angle (such as the case for dynamic soaring seabirds), the solution with the lowest average roll angle will be correct. Importantly, the accuracy of this method relies critically on the quality of the calibration and how well the hard- and soft-iron offsets are accounted for. In addition, the calibration values might have to be adjusted for deployments spanning several days where individuals are subject to varying magnetic field intensities. Future studies could use IMUs that include accelerometers, magnetometers and gyroscopes to estimate accurate body angles. These data could then be used to determine the efficacy of the methods described in the present study for seabirds ranging in body size and flight modes. We did not have reliable measurements of yaw angles against which to compare our magnetometer derived yaw angles. Yaw angles could potentially be extracted from video data, when the sun is present in a frame and the time and location are known [12], but this is not something we explicitly tested and more research is needed to confirm the accuracy of yaw estimates.

## Data Availability

Data are archived on the University of Cape Town's ZivaHub digital repository at: https://doi.org/10.25375/uct.21312678. R code used to run the analysis is also available at: https://github.com/sschoombie/albatross_angle.
